# A novel risk‐scoring system for predicting lymph node metastasis of rectal neuroendocrine tumors

**DOI:** 10.1002/ags3.12355

**Published:** 2020-06-10

**Authors:** Keigo Chida, Jun Watanabe, Kingo Hirasawa, Yoshiaki Inayama, Toshihiro Misumi, Chikara Kunisaki, Itaru Endo

**Affiliations:** ^1^ Department of Gastroenterological Surgery Yokohama City University Graduate School of Medicine Yokohama Japan; ^2^ Department of Surgery Gastroenterological Center Yokohama City University Medical Center Yokohama Japan; ^3^ Division of Endoscopy Yokohama City University Medical Center Yokohama Japan; ^4^ Department of Pathology Yokohama City University Medical Center Yokohama Japan; ^5^ Department of Biostatistics Yokohama City University School of Medicine Yokohama Japan

**Keywords:** lymphatic metastasis, neuroendocrine tumor, risk assessment

## Abstract

**Aim:**

Although rectal neuroendocrine tumors (NETs) are considered to be rare low‐grade malignancies when lymph node metastasis (LNM) is present, their degree of malignancy is comparable to that of colorectal cancer (CRC). However, it remains unclear as to which patients require radical lymph node dissection. The aim of this study was to elucidate the risk factors for LNM and develop a risk‐scoring system for LNM to help determine appropriate therapeutic approaches.

**Methods:**

In this study, we examined 103 patients with rectal NETs who underwent local resection (n = 55) or radical resection with LN dissection (n = 48). We evaluated each pathological feature, including the depth of submucosal invasion (SM depth) and tumor budding grade.

**Results:**

According to our univariate analyses and previous reports, the significant five risk factors for LNM were weighted with point values: 2 points for tumor size ≥ 15 mm and muscularis invasion, and 1 point each for SM depth ≥ 2000 µm, positive lymphovascular invasion, budding grade 3, and vertical margin. The area under the receiver operating curve for the scoring system was 0.899 (95% CI: 0.843‐0.955). When a score of 2 was used as the cut‐off value, the sensitivity and specificity for the prediction of LNM were 100% and 72.1%, respectively.

**Conclusions:**

The risk‐scoring system for LNM of rectal NETs showed high diagnostic performance. Using this risk‐scoring system, it is possible to predict the risk of LNM and thereby potentially avoid unnecessary surgery. Further prospective external validation studies should be performed. The study was registered in the Japanese Clinical Trials Registry as UMIN000036658.

## Graphical abstract

The risk‐scoring system for LNM of rectal NETs could predict the risk of LNM and thereby potentially avoid unnecessary surgery.

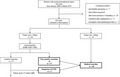

## 1 INTRODUCTION

Rectal neuroendocrine tumors (NETs) are rare malignancies; however, their incidence is increasing, which may reflect the incidental identification of lesions due to the increased availability of colonoscopy and radiological imaging.^1^, ^2^, ^3^ Though rectal NETs are generally indolent and have good prognosis, several reports indicated that the long‐term prognosis of rectal NETs with lymph node metastasis (LNM) is comparable to that of colorectal cancer (CRC).^4^, ^5^ Accordingly, various studies have reported different predictors of LNM, including a tumor size of >10 mm or >20 mm, muscle invasion, lymphovascular invasion (LVI), or tumor grade^3^, ^4^, ^6^, ^7^, ^8^, ^9^; however, the risk factors for LNM have not been clearly elucidated. As a result, there have been disputes about the treatment strategy for rectal NETs, especially as to whether tumors of 10 to 20 mm in size require radical LN dissection or whether they can be treated with local resection.^4^, ^10^, ^11^, ^12^ Hence, it is crucial to evaluate the predictors of LNM in patients with rectal NETs using more detailed clinicopathological data.

In early CRC, the depth of submucosal invasion (SM depth) and tumor budding grade are regarded as strong predictors of LNM.^13^, ^14^ In Japan, these factors are considered in the therapeutic strategy for early CRC.^15^ However, few studies on rectal NET have focused on these factors, which are usually evaluated as predictors of LNM in CRC. As rectal NETs arise from the deep portion of glands and are mainly localized in the SM layer,^16^, ^17^ we suggest these variables should be evaluated in rectal NETs as they are in early CRC.

If the patients with a low risk of LNM can be identified, the wider application of local resection without additional treatment may be an acceptable option. Thus, the aim of this study was to evaluate the risk factors for LNM in patients with rectal NETs based on detailed clinicopathological features and to develop a risk‐scoring system for LNM.

## 2 METHODS

**FIGURE 1:**
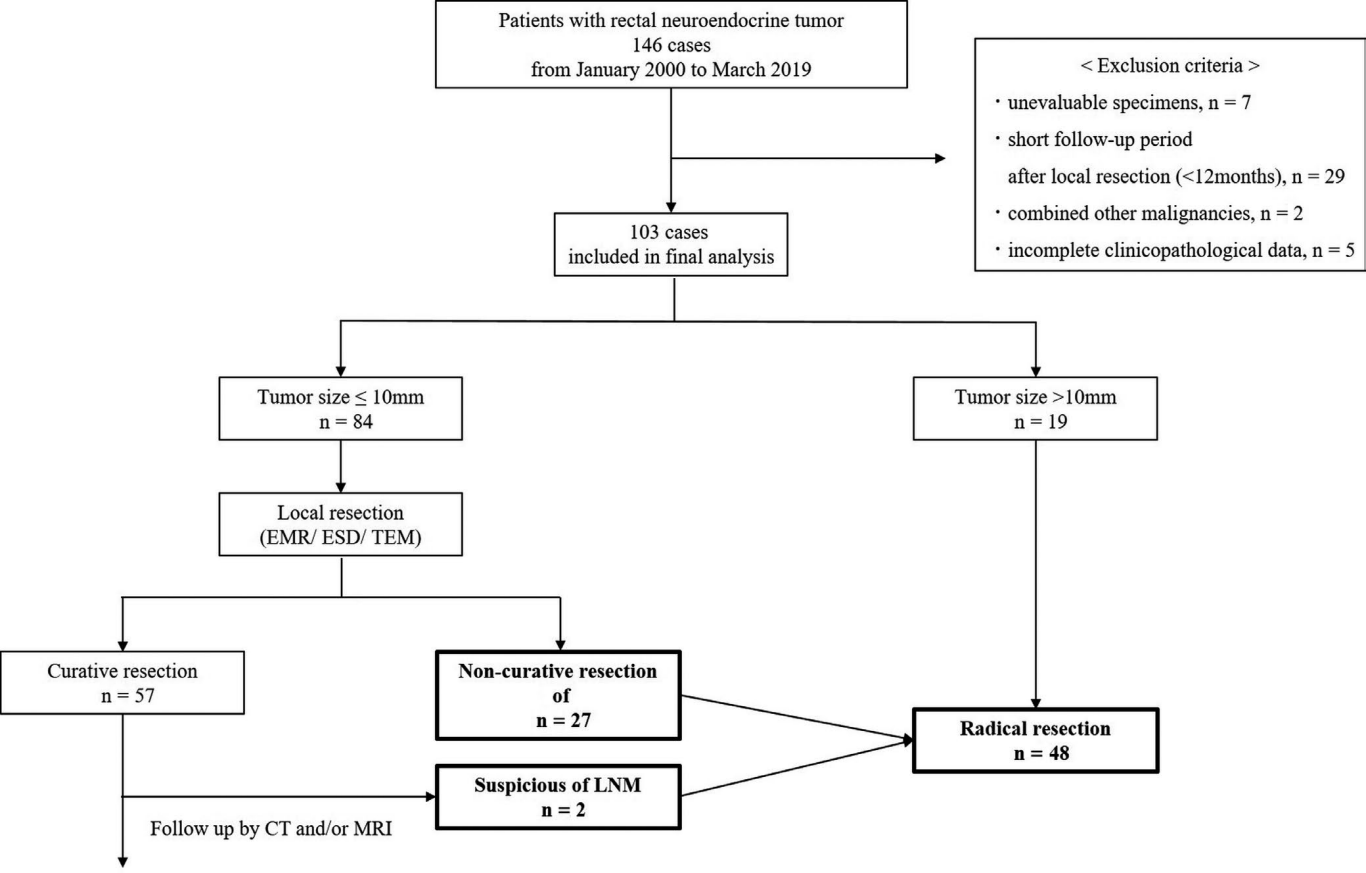
Flow chart of the study cohort

The study protocol was approved by the Ethical Advisory Committee of Yokohama City University Medical Center. The study was registered with the Japanese Clinical Trials Registry as UMIN000036658 (http://www.umin.ac.jp/ctr/index.htm). The study population included a total of 144 patients with rectal NETs who underwent local resection (endoscopic mucosal resection [EMR], endoscopic submucosal dissection [ESD], or transanal excision [TEM]) and surgical resection at Yokohama City University Medical Center between 1 January 2000 and 31 May 2019. The exclusion criteria were as follows: patients with unevaluable specimens, other combined malignancies, <12 months of follow‐up after local resection, and incomplete clinicopathological data (Figure 1).

### 2.1 Clinicopathological features

The following variables were obtained from hospital records: age, sex, tumor location within the rectum, distance from the anal verge, and endoscopic maximum tumor diameter were obtained. Tumor location was classified according to distance from the anal verge as follows: upper rectum (>10 cm), middle rectum (5‐10 cm), and lower rectum (<5 cm). All specimens were referred to certified pathologists and the tumor size, depth of invasion, LVI, resection margin status, World Health Organization (WHO) tumor grade, and budding grade were evaluated.^18^ The diagnosis of rectal NET was confirmed by hematoxylin‐eosin (HE) staining and immunohistochemical staining (synaptophysin, chromogranin A, and CD56). The tumor grade was classified according to the WHO classification 2010.^19^ The mitosis count was expressed as the number of mitotic cells per high‐power field (with HE‐staining), while the Ki‐67 index was calculated as the percentage of cells labeled by immunohistochemical staining.

**FIGURE 2:**
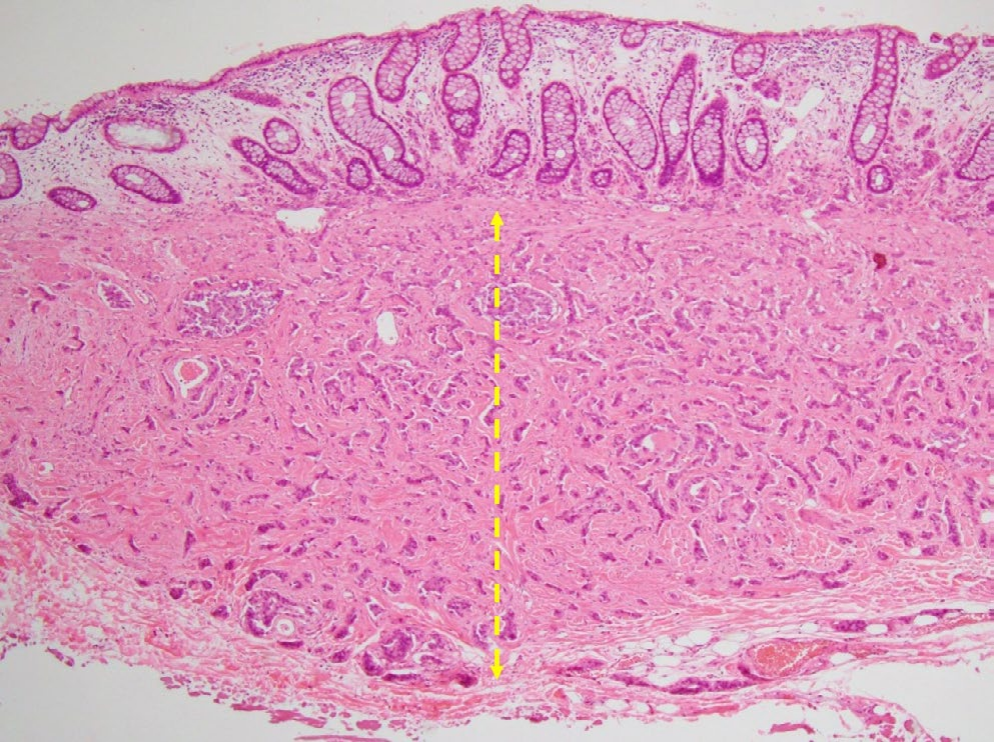
HE‐staining of the identified muscularis mucosa. The SM depth is indicated by dot yellow arrow

**FIGURE 3:**
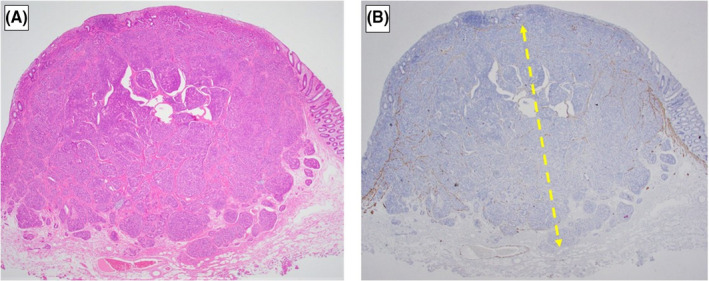
*HE (A) and Desmin (B) staining of the non‐identified muscularis mucosa*

All specimens were subjected to D2‐40 and Elastica van Gieson staining and LVI was considered positive when tumor cells were present within the vascular spaces lined by stained endothelial cells. Since the method for setting a baseline for the SM depth of rectal NETs is not defined, we measured them according to the criteria for nonpedunculated submucosally invasive CRC.^13^ However, in rectal NETs, the muscularis mucosae—as baselines—is unclear in some cases because they arise from the bottom of mucosa and the rupture of muscularis mucosae is often observed due to their invasion. Thus, in the cases without identifiable muscularis mucosae, we set the baseline using Desmin staining (HE‐staining of the identified muscularis mucosa [Figure 2]; HE and Desmin staining of the non‐identified muscularis mucosa [Figure 3A,B]). The SM depth was determined by microscopic observation of specimens using an optical micrometer. Moreover, since there is also no definition of the budding grade of rectal NETs, we evaluated them as we would CRC.^14^

**FIGURE 4:**
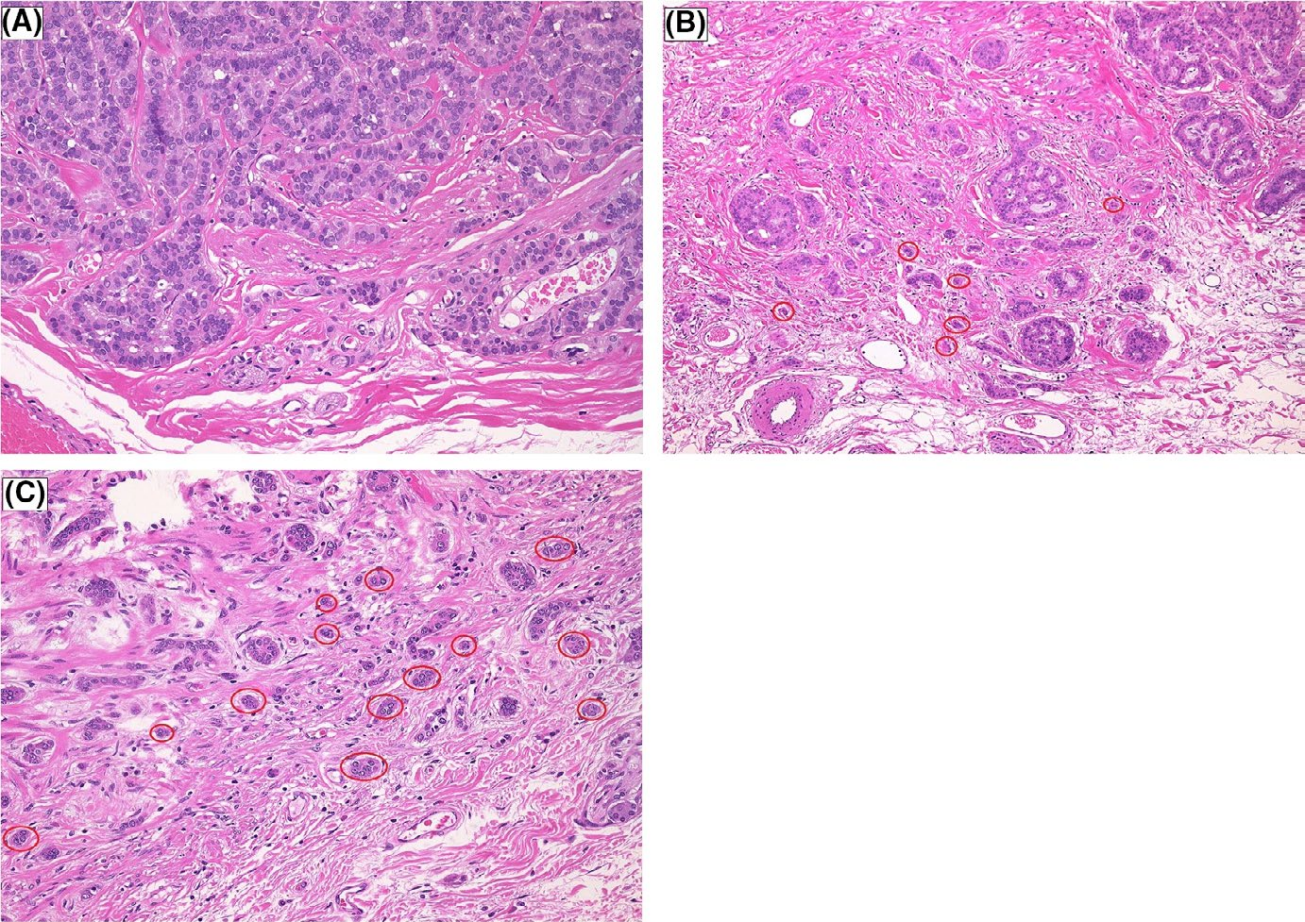
Examples of tumor budding grade at the invasive front of the rectal NET in the selected hot spot (20× objective). Tumor budding is circled by a red ring around. A, Budding grade 1 (low), B, Budding grade 2 (moderate), C, Budding grade 3 (high)

In the colorectal cancer, tumor budding is defined as a single tumor cell or a cell cluster of up to four tumor cells. In the rectal NETs, we selected the HE‐slide with the greatest degree of budding at the invasive front and scanned individual fields at medium power (10× objective) to identify the “hotspot”. Then, we counted tumor budding in the selected “hotspot” (20× objective) and selected the budding category based on the budding count according to the definition of colorectal cancer. We classified tumor budding grade in the rectal NETs according to ITBCC criteria: budding grade 1 (low): 0‐4 budding counts; budding grade 2 (intermediate): 5‐9 budding counts; budding grade 3 (high): 10 or more budding counts^14^ (Figure 4A,B,C).

### 2.2 Endoscopic procedure

Small rectal NETs of ≤10 mm in size without MP invasion or LMN before surgery have principally been treated by local resection. Endoscopic mucosal resection with a ligation device (EMR‐L) or endoscopic submucosal dissection (ESD) were performed. Basically, we have chosen ESD for rectal NETs due to the advantage for resectability and curability compared with EMR‐L.^20^

Curative resection criteria for endoscopic resection (ER) were the following: (i) negative resection margins; and (ii) negative LVI after local resection. In addition, we render NET: G2 as the non‐curative resection for ER due to its higher risk of LNM.^9^ Additional radical resection was performed for the cases that didn't meet curative criteria for ER and for the curative resection cases with swollen lymph nodes detected during follow‐up period by computed tomography (CT) and/or magnetic resonance imaging (MRI). The definition of the swollen lymph node was described later. These indications for radical resection were similar to those of the 2015 Japan neuroendocrine tumor society clinical practice guidelines for pancreatic Gastrointestinal‐NET.^12^

### 2.3 Surgical procedures

Surgical resection for rectal NETs included total mesorectal excision (TME) or tumor‐specific mesorectal excision (TSME). The level of ligation of the inferior mesenteric artery depended on the preoperative diagnosis and the decision of the treating surgeons. Lateral lymph node dissection was not performed. All laparoscopic operations were carried out or supervised by surgeons qualified under the Endoscopic Surgical Skill Qualification System of the Japan Society for Endoscopic Surgery.^21^

### 2.4 Follow‐up surveillance strategy

During the follow‐up period, the lymph nodes were assessed by CT or MRI of the abdomen and pelvis. Lymph node metastasis was defined as positive if CT or MRI revealed nodes of >3 mm in diameter in the perirectal area or nodes of >10 mm in diameter in the pelvis.^22^

### 2.5 Statistical analyses

Quantitative data are expressed as the median and interquartile range (IQR). We used the Mann‐Whitney U test to compare the continuous variables (such as age) and Fisher's exact probability test to compare the proportions of categorical variables (such as sex). *P* values of <.05 were considered to indicate statistical significance. Receiver‐operating characteristic (ROC) curves were plotted, and the areas under the ROC curve (AUC) were determined. In addition, for further internal validation of our scoring system for the LNM, the estimates of AUC and 95% confidence interval (CI) were calculated with 1000 replications of bootstrap samples in our cohort. All statistical analyses were performed using EZR (Saitama Medical Center, Jichi Medical University, Saitama, Japan), which is a graphical user interface for R (The R Foundation for Statistical Computing, Vienna, Austria). More precisely, it is a modified version of R commander designed to add statistical functions frequently used in biostatistics.

## 3 RESULTS

### 3.1 Clinicopathological and operative factors of rectal NETs

A total of 103 patients were analyzed in this study. After local resection, non‐curative resection cases (n = 27) received radical resection. In the cases that met curative resection criteria, two cases were suspicious of the LNM during follow‐up period and they were performed by radical resection. Median follow‐up period of curative resection cases was 41 months (range: 12‐194). Table 1 shows the baseline clinicopathological characteristics of the rectal NETs. The incidence of LNM was 16.5% (17/103). Table 2 shows the operative factors. In almost all cases, surgery was performed laparoscopically, and lymph node dissection was adequate.

**TABLE 1:** Baseline clinicopathological characteristics

Clinicopathological factors	Total n = 103	
	n	IQR or %
Age, year (IQR)	56	(45‐65)
Sex, n (%)		
Male	63	61.2%
Female	40	38.8%
Size, mm (IQR)	7	(5.0‐9.5)
Depth of invasion		
sm	100	97.1%
mp	3	2.9%
SM depth, μm (IQR)	2225	(1300‐4000)
Distance from anal verge, cm (IQR)	5	(4.0‐6.0)
Location of rectum, n (%)		
Upper	31	30.1%
Middle	66	64.1%
Lower	6	5.8%
Lymphovascular invasion, n (%)		
Positive	33	32.0%
Negative	70	68.0%
Vertical margin, n (%)		
Positive	13	12.6%
Negative	71	68.9%
WHO classification, n (%)		
Grade 1	95	92.2%
Grade 2	8	7.8%
Budding Grade, n (%)		
Grade 1	92	89.3%
Grade 2	7	6.8%
Grade 3	4	3.9%
LNM, n (%)	17	16.5%

Note

Variables are n (%) or mean (interquartile range: IQR), unless otherwise indicated.

Abbreviations: LNM, lymph node metastasis; LVI, lymphovascular invasion; mp, muscularis propria; Sm, submucosa; VM, vertical margin.

**TABLE 2:** Operative factors

Treatment factors	Total n = 103	
	n	IQR or %
Therapeutic method, n (%)		
Curative local resection	55	53.4%
Additional radical resection	29	28.2%
Non‐curative local resection^a^	27	
Regional lymph node recurrence after curative local resection	2	
Radical resection	19	18.4%
Local resection, n (%)		
EMR	13	12.6%
ESD	37	35.9%
TEM	5	4.9%
Operative procedure, n (%)		
Open‐LAR	1	1.0%
Lap‐LAR	9	8.7%
Lap‐vLAR	26	25.2%
Lap‐ISR	12	11.7%
Lymph node dissection, n (%)		
D1	2	1.9%
D2	18	17.5%
D3	28	27.2%
Operative time, min (IQR)	225	(189‐227)
Blood loss, g (IQR)	20	(20‐121)
Number of dissected lymph node (IQR)	17	(11‐20)

Note

Variables are n (%) or mean (interquartile range: IQR), unless otherwise indicated.

Abbreviations: APR, anterior peritoneal resection; EMR, endoscopic mucosal resection; ESD, endoscopic submucosal dissection; ISR, intersphincteric resection; LAR, low anterior resection; TEM, transanal endoscopic microsurgery; vLAR, very low anterior resectio.

^a^ After local resection, we performed the radical resection to the patients who had Lymphovascular invasion (+), muscularis invasion (+), vertical margin (+) and/or NET:Grade2.

The univariate analysis of risk factors for lymph node metastasis and the establishment of a risk‐scoring system to predict LNM. In the univariate analysis, endoscopic tumor size, SM depth (≥2000 μm), LVI (+), and budding grade 3 were significantly associated with LNM. No LNM was observed in cases with an SM depth of <2000 μm (Table 3). Furthermore, regarding the relationship between budding grades 1‐3 and each pathological factor, there were no significant differences except for in the WHO classification (Table S1).

**TABLE 3:** The univariate analysis of risk factors for lymph node metastasis

Clinicopathological factors	Lymph node metastasis				*P* value
	Negative n = 86		Positive n = 17		
	n	IQR or %	n	IQR or %	
Age, year (IQR)	56	(46‐65)	57	(45‐64)	1.000
Sex					
Male	52	60.5%	11	64.7%	
Female	34	39.5%	6	35.3%	
Size, mm (IQR)	6.5	(5.0‐9.0)	9.0	(7.0‐15.0)	<0.001
Size, mm					
<15	81	94.2%	12	70.6%	0.010
≥15	5	5.8%	5	29.4%	
Depth of invasion					
sm	84	97.7%	16	94.1%	0.421
mp	2	2.3%	1	5.9%	
SM depth, μm (n = 100)	(n = 84)		(n = 16)		<0.001
<2000	45	53.6%	0	0.0%	<0.001
≥2000	39	46.4%	16	100.0%	
Location of rectum					
Upper	5	5.8%	1	5.9%	0.905
Middle	56	65.1%	10	58.8%	
Lower	25	29.1%	6	35.3%	
Lymphovascular invasion, n (%)					
Positive	20	23.3%	13	76.5%	<0.001
Negative	66	76.7%	4	23.5%	
Vertical margin, n (%)					
Positive	9	12.0%	3	33.3%	0.115
Negative	66	88.0%	6	66.7%	
WHO classification, n (%)					
Grade 1	80	93.0%	15	88.2%	0.616
Grade 2	6	7.0%	2	11.8%	
Budding Grade, n (%)					
Grade 1/2	85	98.8%	14	82.4%	0.011
Grade 3	1	1.2%	3	17.6%	

Note

Variables are n (%) or mean (interquartile range: IQR), unless otherwise indicated.

Abbreviations: LVI, lymphovascular invasion; mp, muscularis propria; sm, submucosa; VM, vertical margin.

Based on the results of the univariate analysis and previous studies, we established a risk‐scoring system to predict a patient's risk of developing LMN. We adopted endoscopic tumor size ≥ 15 mm, LVI (+), SM depth (≥2000 μm), and budding grade 3 as predictive variables to develop the clinical risk score for radical resection (including additional resection). In addition, based on previous studies, we included MP invasion, tumor grade G2, and positive VM as potential risk factors for LNM.^6^, ^7^, ^9^, ^23^ To calculate the risk score, we assigned points for each of the seven predictive variables: 1 point each for LVI (+), SM depth ≥ 2000 µm, budding grade 3, tumor grade G2 and positive VM respectively, and 2 points each for endoscopic tumor size ≥ 15 mm, and MP invasion. Regarding tumor size ≥ 15 mm and MP invasion, we assigned a higher score in view of the results of previous reports.^4^, ^6^, ^24^, ^25^, ^26^ The total risk score, which ranged from 0 to 9 points, was calculated for each patient by adding the points for each of the patients’ risk factors. Table 4 shows the incidence of LNM according to each score. The scoring system yielded an AUC of 0.899 (95% CI 0.843‐0.955) for LNM. This scoring system tended to show a higher predictive ability for LNM than the conventional strategy, which has traditionally been used in Japan, based on the Japanese clinical practice guideline for gastrointestinal NET (AUC: 0.899 vs 0.821, *P* = .059).^12^ When the cut‐off value of the score was set at 2, the sensitivity and specificity in the prediction of LNM were 100% and 72.1%, respectively. For the internal validation of the scoring system, 1000 bootstrapped replications were performed to resample the data. The results of the bootstrapping analysis were similar to those obtained with the original samples (AUC:0.898, 95% CI, 0.840‐0.947).

**TABLE 4:** The rate of lymph node metastasis rate for each score

Total points	Total cases n = 103	LNM n = 17	Rate of LNM (%)
0	33	0	0%
1	29	0	0%
2	25	7	28%
3	8	6	75%
4	5	2	40%
5	3	2	67%

Note

Variables are n (%) or mean (interquartile range: IQR), unless otherwise indicated.

Abbreviation: LNM, lymph node metastasis.

## 4 DISCUSSION

The purpose of this study was to elucidate the risk factors for LNM in patients with rectal NETs and to develop a risk‐scoring system. We found that endoscopic tumor size, LVI, SM depth ≥ 2000 μm, and budding grade 3 were risk factors for LNM in a univariate analysis. However, the prediction for LNM by SM depth (<2000 µm vs ≥2000 µm) was statistically defined as complete separation. Complete separation occurs when a linear combination of predictors provides a complete prediction of the response variable. In the case of complete data separation, maximum likelihood estimates for the multivariate logistic regression do not exist. Thus, we developed a scoring system in which each factor was weighted in reference to previous reports. Each score was determined as follows.

- Depth of invasion: Generally, in cases of rectal NETs, MP invasion is considered an absolute indication for surgery due to the high LNM rate.^6^, ^7^ This is reasonable, considering the frequency of LNM of MP invasion in colorectal cancer.^27^ However, although the SM depth was not evaluated in the rectal NETs in the present study, we did identify an SM depth ≥ 2000 µm as a risk factor for LNM for the first time. Therefore, given that MP invasion is considered an absolute indication for surgery, we decided to assign half of that point to SM ≥ 2000 µm in the present study.
- Tumor size: Rectal NETs greater than 10 mm have been considered an absolute surgical indication due to the associated high LNM rate. Several studies have suggested that rectal NETs of 10 to 15 mm in size can be treated endoscopically if they did not have proper muscle invasion or LNM.^26^, ^28^ Therefore, endoscopic resection should be considered for most rectal NETs smaller than 10 mm and can be considered for rectal NETs 10 to 15 mm in size. In the present study, LNM was detected in 5 out of 10 patients (50%) who had tumors > 15 mm, but no LNM was observed in cases with a tumor diameter of 10‐14 mm without other predictive factors. Accordingly, we decided to assign tumor size ≥ 15 mm the same points as MP invasion, which is an absolute surgical indication.
- LVI: Because many previous studies have reported that the LVI is a risk factor for metastasis in rectal NETs,^26^, ^29^ rectal NETs with LVI have been considered for surgical indication. However, Sekiguchi et al suggested that the presence of LVI may not clearly indicate the risk of metastasis.^8^ Indeed, in the present study, five cases with only LVI as a risk factor did not have LNM. Therefore, we decided to assign LVI a half a point compared to MP invasion, which is an absolute surgical indication.
- VM and NET G1 or G2 (Ki‐67): VM and Ki‐67 have been considered as risk factors for LNM, but the precise risk for LNM is controversial in cases of rectal NETs.^30^ Therefore, we decided to assign VM positivity and NET G2 status the same number of points as LVI.
- Budding grade: There are no previous reports concerning the budding grade for LNM in rectal NETs. Our results suggested that budding grade 3 was associated with LNM in rectal NETs. Given the small number of budding grade 3 cases, we decided to assign budding grade 3 the same number of points as LVI.

We understand that this scoring approach is arbitrary, but we consider this method to be the best, given the current number of cases and previous reports. In the future, the accuracy of this scoring system should be externally validated. We are now planning a multi‐center study to validate this scoring system.

This scoring system had a high diagnostic performance. When we performed radical resection in cases with a score of ≥2, the sensitivity and specificity for predicting LNM were 100% and 72.1%, respectively. This meant that radical resection could have been avoided in a total of 13 cases in which it would have been indicated according to our conventional therapeutic strategy. Originally, SM depth ≥ 1000 µm was considered to be a risk factor for LNM in patients with early CRC.^13^ Although almost all rectal NETs are confined to the submucosa layer, the relationship between SM depth and LNM has seldom been evaluated. Lee et al examined the classified SM depth and the association of LNM and reported that pT1b or deeper was significantly associated with LNM.^22^ However, that report might not have been able to reveal the true risk of LNM due to the small number of patients who underwent surgery (n = 13). Moreover, the criteria for the measurement of the SM depth were not explicit. It is imperative to set the baselines because the muscularis mucosae of rectal NETs are unclear in some cases due to tumor invasion. Therefore, we set the baseline by using Desmin‐staining to clarify it in those cases. As a result, SM ≥ 2000 µm was found to be significantly associated with LNM in this study.

The tumor budding grade is an essential predictor of LNM in early CRC^31^, ^32^; however, it has not been clear whether it is associated with the risk of LNM in patients with rectal NETs. Even though the association between the histological pattern of rectal NETs and LNM was reported previously,^22^, ^33^ no reports have focused on the morphological features at the invasive front of the tumor. In this report, we examined the association between structural atypia at the invasive front of rectal NETs and LNM, according to the tumor budding grade.^14^ In the univariate analysis, budding grade 3 was significantly associated with LNM.

Moreover, many previous studies have reported that the presence of LVI is a risk factor for metastasis in patients with rectal NETs.^26^, ^29^ However, Sekiguchi et al reported that when a histological evaluation is performed using immunohistochemical staining, almost half of small rectal NETs were positive for LVI but none of the patients developed recurrence. Based on these results, they suggested that the presence of LVI may not clearly indicate the risk of metastasis.^8^ Actually, in this study, five cases with only LVI had no LNM. This shows that even LVI, which was considered one of the strongest predictors of LNM, did not have sufficient reliability to indicate radical resection. We hypothesized that the selection of therapeutic strategies based on a single pathological finding could lead to overtreatment. Since most rectal NETs exist in the lower rectum, higher surgical complications and impaired quality of life, including fecal incontinence and dysuria, are matters of concern. We therefore believe that the surgical indications for rectal NETs should be reconsidered.

The present study is associated with several limitations. First, the scoring system was not developed according to the multivariate analysis, as mentioned above. Second, this was a retrospective study and performed in a single institution. Thus, even though we performed internal validation, the statistical analyses of the risk factors for LNM could not determine any cause‐and‐effect relationship. Third, we evaluated the morphological features at the invasive front according to the budding grade. We would have liked to have examined according to the budding grades used for CRC in rectal NETs; however, there was no definition as such. Certainly, tumor budding is defined at the invasive margin of CRC.^31^ It might have been better to have avoided adopting the criteria used for CRC in the assessment of rectal NETs because of their oncologic differences. However, we believe that nothing is more important than the tumor budding when evaluating the invasive front of tumors. Our study suggested that the morphological features at the invasive front could be a useful predictor of LNM in patients with rectal NETs, which warrants a further evaluation in a large cohort. Forth, though our median follow‐up period was 41 months, and this is acceptable compared to the past studies, a longer follow‐up period should be required because some reports demonstrated the recurrent cases after a long period of local resection.^23^ Moreover, because, in the curative local resection cases, the regional lymph node metastasis could not be histologically evaluated, we cannot really omit the micrometastasis in the patients.

In conclusion, the risk‐scoring system for LNM in patients with rectal NETs showed high diagnostic performance, especially with regard to sensitivity. Using this risk‐scoring system, it is possible to predict the risk of LNM and potentially avoid unnecessary surgery. Further prospective external validation studies should be performed to determine the utility of this scoring system.

## DISCLOSURE

Conflict of Interest: The authors declare no conflicts of interest in association with the present study.

## Supporting information

Table S1
