# Supplementary material for: A novel risk‐scoring system for predicting lymph node metastasis of rectal neuroendocrine tumors
Source: Ann Gastroenterol Surg. 2020 Jun 10;4(5):562–70. doi: 10.1002/ags3.12355 (PMC7511567; doi:10.1002/ags3.12355)
Supplement: Supplementary file 1 — Table S1 [file AGS3-4-562-s001.docx]

**Supplementary table.1**

**The relationship between Budding Grade 1-3 and the other pathological factors.**

| Variables | Budding Grade | | | | |  |  |
| --- | --- | --- | --- | --- | --- | --- | --- |
|  | BD1 |  | BD2 |  | BD3 |  | p-value |
| Size, mm (range) | 7.0 | 3.0-20.0 | 8.0 | 5.0-11.0 | 10.0 | 9.0-18.0 | 0.106* |
| Depth of invasion |  |  |  |  |  |  |  |
| sm | 89 | 96.7% | 7 | 100.0% | 4 | 100.0% | 1.000 |
| mp | 3 | 3.3% | 0 | 0.0% | 0 | 0.0% |  |
| SM depth, μm (IQR) | 2200 | 1300-3850 | 1400 | 1050-3850 | 4000 | 2900-  5500 | 0.235* |
| Lymphovascular invasion, n(%) |  |  |  |  |  |  | 0.764 |
| positive | 29 | 31.5% | 2 | 28.6% | 2 | 50.0% |  |
| negative | 63 | 68.5% | 5 | 71.4% | 2 | 50.0% |  |
| Vertical margin**, n(%) |  |  |  |  |  |  | 1.000 |
| positive | 11 | 14.5% | 0 | 0.0% | 0 | 0.0% |  |
| negative | 65 | 85.5% | 6 | 85.7% | 2 | 0.0% |  |
| WHO classification, n(%) |  |  |  |  |  |  |  |
| Grade1 | 87 | 94.6% | 5 | 71.4% | 3 | 75.0% | 0.038 |
| Grade2 | 5 | 5.4% | 2 | 28.6% | 1 | 25.0% |  |

*Kruskal-Wallis test

**The patients with endoscopic resection and additional surgical resection (N=84).
